# Contemporary Evaluation and Clinical Treatment Options for Aortic Regurgitation

**DOI:** 10.3390/jcdd10090364

**Published:** 2023-08-25

**Authors:** Mark Lebehn, Torsten Vahl, Polydoros Kampaktsis, Rebecca T. Hahn

**Affiliations:** 1 Department of Medicine, Columbia University Irving Medical Center, New York, NY 10032, USA; 2 Cardiovascular Research Foundation, New York, NY 10019, USA

**Keywords:** aortic regurgitation, echocardiography, transcatheter aortic valve replacement

## Abstract

Aortic regurgitation (AR) is the third most frequent form of valvular disease and has increasing prevalence with age. This will be of increasing clinical importance with the advancing age of populations around the globe. An understanding of the various etiologies and mechanisms leading to AR requires a detailed understanding of the structure of the aortic valve and aortic root. While acute and chronic AR may share a similar etiology, their hemodynamic impact on the left ventricle (LV) and management are very different. Recent studies suggest current guideline recommendations for chronic disease may result in late intervention and suboptimal outcomes. Accurate quantitation of ventricular size and function, as well as grading of the severity of regurgitation, requires a multiparametric and multimodality imaging approach with an understanding of the strengths and weaknesses of each metric. Echocardiography remains the primary imaging modality for diagnosis with supplemental information provided by computed tomography (CT) and cardiac magnetic resonance imaging (CMR). Emerging transcatheter therapies may allow the treatment of patients at high risk for surgery, although novel methods to assess AR severity and its impact on LV size and function may improve the timing and outcomes of surgical intervention.

## 1. Introduction

Valvular heart disease has been shown to increase in prevalence with advancing age [1,2]. The World Health Organization reports that by 2030, one in six people will be 60 years of age or older (1.4 billion people) [3] and the OxVALVE population Cohort Study of patients ≥ 65 years old found a prevalence of mild aortic regurgitation (AR) of 15% and moderate or severe AR of 1.6% [4]. These findings are in line with the Framingham Offspring Study which found that in patients 60–69 years old, the prevalence of ≥moderate AR was 0.6% in men and 0.8% in women, and between 70 and 83 years the prevalence increased to 2.2% in men and 2.3% in women. A recent community-based study reported a 4.5% prevalence of moderate or severe AR in patients >65 years old [5]. An understanding of the diagnosis and management of AR will thus have increasing clinical importance in the setting of an aging population. 

## 2. Aortic Valve Anatomy

**Figure 1:**
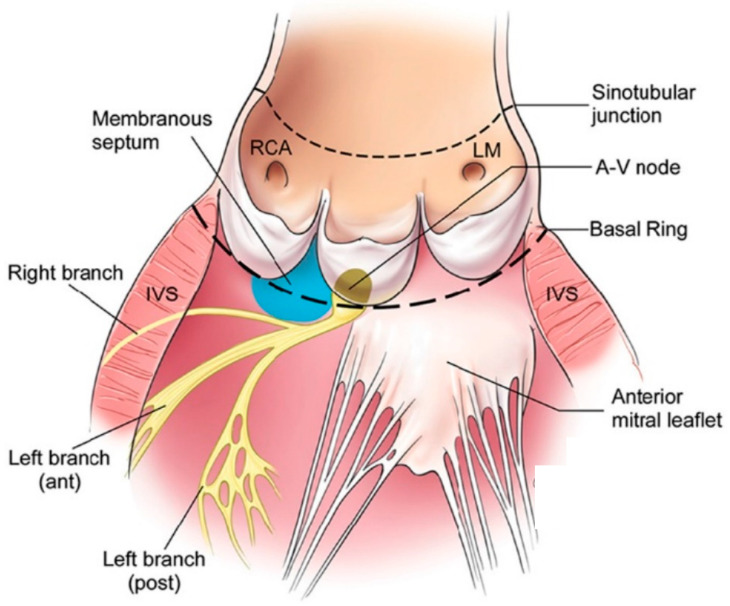
Anatomy of the Aortic Valve. In this figure, the anatomy of the aortic valve complex is shown. The aortic valve is composed of three cusps attached to the root in a semilunar fashion with their nadir of coaptation at the basal ring, representing the level of the annulus, and highest point of attachment of the leaflet commissures at the sinotubular junction. The atrioventricular (AV) node is typically located on the floor of the right atrium just posterior (post) and inferior to the membranous septum (*blue shaded region*). The atrioventricular (AV) node and bundle then courses on the top of the muscular septum under the membranous septum (blue area), where it divides into the left and right bundles. Abbreviations: *ant*, Anterior; *IVS*, interventricular septum; *LM*, left main coronary artery; RCA, right coronary artery. (Reproduced with permission from Hahn RT, Nicoara A, Kapadia S, Svensson L, Martin R. Echocardiographic Imaging for Transcatheter Aortic Valve Replacement. *J. Am. Soc. Echocardiogr.* [6].

**Figure 2:**
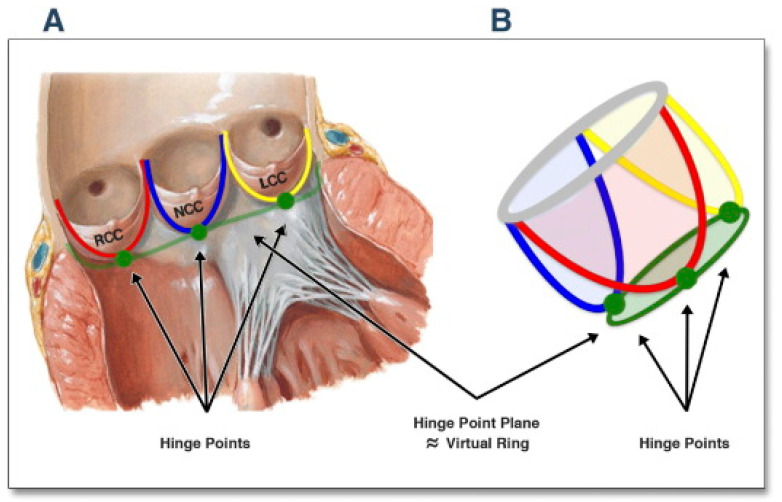
Anatomy of the aortic root. The aortic root is defined as the portion of the aorta between the basal ring within the left ventricle (green line, panel (**A**)) and the sinotubular junction (gray circle, panel (**B**)). The root is described as containing three circular rings: virtual ring at the basal attachments of the leaflets (green line/area), the crown-like ring composed of the semilunar attachments of the leaflets (red-blue-yellow lines) and ring at the level of the STJ. (Reproduced from Kasel AM, Cassese S, Bleiziffer S, Amaki M, Hahn RT, Kastrati A, Sengupta PP. Standardized imaging for aortic annular sizing: implications for transcatheter valve selection. *JACC Cardiovasc. Imaging* [8].

An understanding of the etiologies of AR begins with an understanding of the structure of the aortic valve and aortic root. The aortic valve is composed of three cusps attached to the root in a semilunar fashion with their nadir of coaptation at the level of the annulus and highest point of attachment of the leaflet commissures at the sinotubular junction (STJ) (Figure 1) [6]. The leaflet coaptation surface is only a few millimeters in length. Variation can be seen between leaflets in height, width, surface area, and volume of each of the supporting sinuses of Valsalva [7]. The aortic root is defined as the portion of the aorta between the basal attachment point of the leaflets within the left ventricle (LV) and the STJ. Its components include the leaflets, commissures, interleaflet triangles, STJ, and sinuses of Valsalva. The root is described as containing three circular rings (virtual ring at the basal attachments of the leaflets, ring at the level of ventriculo-arterial junction and ring at the level of the STJ) and a crown-like ring (formed by the suspended leaflets) (Figure 2) [8]. The posterior aspect of the aortic root is supported by fibrous tissue for approximately 55% of its circumference (membranous part of the septum to the left fibrous trigone) while the remainder is supported by ventricular muscle [9].

## 3. Etiologic Classification of Aortic Regurgitation

**Figure 3:**
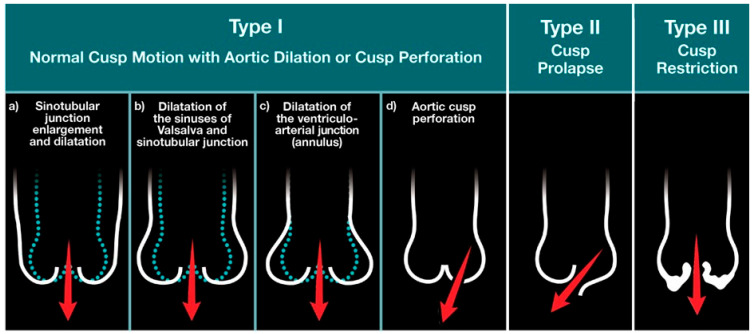
Carpentier Classification of Etiologies of Aortic Regurgitation. Aortic regurgitation can be classified by the etiology of aortic regurgitation as: leaflet malcoaptation/perforation (Type I), excessive leaflet motion (Type II), or restricted leaflet motion (Type III). Type I disease can be further subclassified into the location of the aortic root dilatation: sinotubular junction, sinuses of Valsalva, or ventriculoarterial junction. (After Zoghbi WA, Adams D, Bonow RO, et al. Recommendations for Noninvasive Evaluation of Native Valvular Regurgitation: A Report from the American Society of Echocardiography Developed in Collaboration with the Society for Cardiovascular Magnetic Resonance. *J Am Soc Echocardiogr.* [10]).

In the setting of a trileaflet valve, AR may be caused by primary leaflet disease or abnormalities of the aortic root and ascending aorta. Primary etiologies can be categorized as degenerative, inflammatory, infectious, due to trauma, tissue disruption, iatrogenic, or congenital [10] (Table 1, Supplementary Videos S1–S6). Functionally there can be leaflet prolapse, restriction of leaflet motion, leaflet retraction, fenestration, or perforation [11]. In the surgical literature, a classification scheme constructed after the Carpentier classification for mitral valve disease is used (Figure 3) [10,12]. Leaflet motion can be normal with reduced coaptation due to aorta dilation with central regurgitation (Type I), excessive leaflet motion (Type II), or restricted leaflet motion (Type III) [13]. Rheumatic heart disease remains the leading cause of AR in many low- to middle-income countries [14]. In high-income countries, it is bicuspid aortic valves or secondary to primary disease of the ascending aorta or the sinuses of Valsalva [14].

Diseases of the aortic root and ascending aorta include idiopathic aortic root dilatation, systemic hypertension, annuloaortic ectasia, autoimmune disease (systemic lupus erythematosus, ankylosing spondylitis, Reiter’s syndrome), connective tissue disease (Loeys–Deitz, Marfan syndrome, Ehlers–Danlos, osteogenesis imperfecta), aortitis (syphilitic, Takayasu’s arteritis), aortic dissection, trauma (Table 1) [10,15]. The 2022 ACC/AHA Guideline for the Diagnosis and Management of Aortic Disease provides recommendations on the timing of imaging, timing of intervention depending upon aortic size in specific disease states, and recommendations on indexing to the patients’ BSA or height [16]. With an aortic dissection, AR and poor leaflet coaptation can result from: dilation of the root and annulus, propagation of a false lumen leading to leaflet prolapse/flail, or a dissection flap prolapsing across the valve [10]. 

Mixed etiologies of regurgitation and mixed aortic valve disease (MVAD) should be considered when interrogating the aortic valve. In a large retrospective series of patients referred for surgical aortic valve replacement or repair, Yang et al. found the most common form of mixed etiologies was prolapse with aortic root dilation in both patients with bicuspid and tricuspid aortic valves [11]. A nationwide epidemiology study in Sweden by Andell et al. found 17.9% of patients with AR had concomitant aortic stenosis (AS) and MVAD was the most frequent form of mixed valve disease [17,18]. Co-existing AS is important to note as studies have shown the prognosis of combined moderate AS and moderate AR is similar or worse than with either isolated AS or AR [17,19,20]. 

## 4. Natural History of Aortic Regurgitation

Differences are found in the natural history and presentation of patients with bicuspid versus tricuspid valves with AR. Patients with bicuspid valves have been found to present earlier and more frequently have mixed mechanisms of AR [11,21,22,23]. In a large contemporary cohort of 798 patients comparing bicuspid and tricuspid aortic valve patients with AR, Yang et al. found bicuspid patients presented two decades younger, underwent aortic valve surgery more frequently with fewer complications, and had a robust association between baseline symptoms and chamber remodeling [21]. In bicuspid patients, mortality risk increases in patients over 50–55 years of age. After adjusting for age, survival was found to be similar between bicuspid and tricuspid valve patients [21].

### 4.1. Acute Aortic Regurgitation

Acute AR can lead to a negative spiral of effects due to acute volume overload to a left ventricle (LV) unable to compensate acutely with chamber dilation. This contrasts with chronic AR where compensatory remodeling allows the patient to remain compensated for years or decades despite the progressing severity of regurgitation. Acute volume overload from AR leads to a rapid rise in left ventricular end-diastolic pressure (LVEDP) and if untreated can lead to hemodynamic compromise. The acute rise in LVEDP leads to a rapid decrease in the gradient across the mitral valve into the LV. Reverberation, reverse doming of the anterior mitral valve leaflet, and premature mitral valve closure can be seen. If LVEDP exceeds left atrial pressure, mitral regurgitation can occur in systole or diastole. Grading of the timing of the mitral valve closure can give an indication of the severity of acute AR and elevation of LVEDP [24]. Further upstream sequelae include congestion with elevation of left atrial pressure and pulmonary edema. Negative effects on the LV from AR are twofold. As LVEDP approaches aortic diastolic pressure, subendocardial hypoperfusion can occur due to a drop in LV myocardial perfusion pressure. In addition to the volume overload, there is an increase in afterload which leads to increased systolic wall stress. Distention of the LV and dilation of the mitral valve annulus can lead to secondary mitral regurgitation. As the LV is unable to acutely increase cardiac output, there is a compensatory increase in heart rate to maintain cardiac output. Acute regurgitation signs and symptoms can include shortness of breath, tachycardia, chest discomfort, peripheral signs of hypoperfusion, and pulmonary congestion. Physical signs in acute AR are less pronounced compared with chronic severe AR. 

### 4.2. Chronic Aortic Regurgitation

With chronic AR, compensatory LV remodeling occurs in response to chronic pressure and volume overload. To maintain forward stroke volume there is an adaptive increase in LV end-diastolic volume and increase in LV compliance to maintain a normal LVEDP. The degree of LV dilation in chronic isolated AR reflects the duration and severity of disease. LV wall stress rises due to increased afterload from increased systolic blood pressure and increasing LV chamber volume [25]. A combination of concentric and eccentric hypertrophy ensues. A reduction in ejection fraction occurs when preload reserve is exceeded, and hypertrophic changes are unable to meet afterload excess [26,27,28]. In chronic severe AR, end-systolic wall stress can be as high as in aortic stenosis [29,30]. In patients with MVAD, LV remodeling due to AS includes concentric remodeling/hypertrophy with or without replacement fibrosis. These changes reduce LV compliance and the ability of the LV to tolerate increasing LV end-diastolic volume from AR. 

Chronic AR is defined by Stages A-D, from at risk of developing AR (Stage A) to severe symptomatic AR (Stage D) [14]. The likelihood of progression to symptoms or LV dysfunction with chronic AR averages 4–5% per year and the average mortality rate is less than 0.2% per year [25,26].

## 5. Quantification of Disease Severity

In both acute and chronic AR, transthoracic echocardiography (TTE) is the primary imaging modality for diagnosis. The morphology of the leaflets and aortic root, mechanism and severity of regurgitation, and LV size, geometry and function should be delineated. Quantitative, semi-quantitative and qualitative techniques should be integrated when grading the severity (Table 2) [10]. Standardized protocols with use of 2D, 3D, pulsed, color and continuous wave Doppler should be used and supporting guidelines are available [10,14,31,32]. Supportive findings of severe chronic AR include flail leaflet or wide coaptation gap, dilated left ventricle, pressure half-time < 200 msec, prominent holodiastolic flow reversal in the abdominal aorta, dense continuous wave signal, and large color flow convergence [10]. A multiparametric approach is best as there are inherent limitations in each technique and the diagnosis should not be made by a single parameter. Pitfalls in the assumption of LV adaptive changes can occur with chamber dilation due to another cause, an unknown starting cavity size to compare changes to, and failure to normalize to body size. In asymptomatic patients’ accurate assessment of LV, chamber size and LV systolic function become critical as they serve as indicators for the timing of intervention. Stress echocardiography can be used to assess functional capacity, presence of coronary artery disease, and response of LV size and function to exercise [10]. 

Transesophageal echocardiography (TEE) allows for better visualization of valve morphology and can further define quantitative and qualitative parameters needed to confirm AR severity [33]. TEE is recommended in the evaluation for endocarditis with prosthetic valves, in the evaluation for a root abscess, or in the evaluation for aortic injury or dissection. 

Specific guidelines are available for pre-procedural screening and post-intervention assessment in both surgical aortic valve replacement (SAVR) and transcatheter aortic valve replacement (TAVR) [33,34,35]. Similar echocardiographic parameters are used to grade prosthetic aortic regurgitation after TAVR with the use of additional tools including aortography, structural parameters of the valve, and invasive hemodynamic parameters [34]. Systematic use of intraoperative TEE is recommended at key points post procedure to guide decision making post aortic valve repair, aortic valve replacement, and aortic surgery [35].

### 5.1. Multimodality Imaging

Computed tomography (CT), cardiac MRI (CMR), and cardiac catheterization can provide supplemental information in cases where TTE or TEE images are suboptimal or there is a discrepancy between clinical and echocardiographic findings [14,36]. CMR is the reference standard for evaluating cardiac volumes, mass, and systolic function. CMR should be considered in patients with poor visualization of endocardial borders on echocardiography. CMR can provide supplemental information regarding the morphology of the aortic valve, aortic root and thoracic aorta, quantification of AR severity, and tissue characterization in the evaluation for underlying cardiomyopathy. CMR may be useful in special populations (e.g., competitive athletes) to discern pathological cardiac remodeling in the setting of AR from an athletic heart phenotype [37]. Cardiac CT similarly can provide supplemental information on aortic valve morphology and confirm thoracic aorta dimensions and the evaluation for aortic dissection. CT imaging is the primary imaging choice in the evaluation for aortic dissection.

### 5.2. Novel Imaging Parameters

Non-invasive methods for imaging the myocardium may detect early mechanical dysfunction and fibrosis, which may inform the timing of intervention [38]. Speckle-tracking echocardiography for the assessment of ventricular longitudinal strain may be more sensitive in detecting structural changes in the myocardium compared to other conventional parameters such as LVEF and has been useful in the risk stratification of patients with AR [39,40]. LV global longitudinal strain and LV myocardial work indices can provide additional insight into LV function in patients with preserved LV ejection fraction [41,42,43,44,45]. In patients with moderate or severe AR, the presence of scars detected by late gadolinium enhancement on CMR is associated with a 2.5-fold increase in death, and aortic valve replacement was independently associated with a lower risk of mortality [46]. Ventricular arrhythmia in aortic valve disease is commonly found when ambulatory monitoring is performed and can be a marker of the impact of aortic regurgitation on the left ventricular myocardium [47,48].

## 6. Medical and Surgical Management

### 6.1. Medical Management

Acute AR requires rapid diagnosis and treatment as medical management is limited. Medical therapy includes medications to reduce LV afterload. In patients with acute AR due to endocarditis or aortic dissection who are surgical candidates, surgery should not be delayed if there is evidence of hypotension, pulmonary edema, or low cardiac output [14,49,50,51,52]. Prior series have shown that patients undergoing emergent aortic valve surgery for acute AR had low operative mortality and good long-term results [53,54,55,56]. 

In chronic AR, medical management includes treatment of systolic blood pressures >140 mmHg [14]. In severe AR with LV dysfunction with or without symptoms, treatment with Ace inhibitors, ARBs and/or sacubitril/valsartan is recommended in prohibitive surgical risk patients [14]. The interval timing of repeat echocardiograms to monitor for progression is every 3–5 years with mild AR, every 1–2 years with moderate AR, and every 6–12 months with severe AR. In chronic severe AR, surgery is indicated in symptomatic patients regardless of LV systolic function. In asymptomatic severe regurgitation, surgery is indicated with LV systolic dysfunction (LVEF ≤ 55%) without another identifiable cause. SAVR is recommended in moderate or severe AR in patients undergoing cardiac surgery for another indication. SAVR is also recommended for asymptomatic severe patients with normal LV systolic function (LVEF > 55%) with severe LV enlargement (LVESD > 50 mm, LVESD > 25 mm/m^2^), or in low surgical risk patients with progressive decline in LVEF (to <55–60%) or progressive increase in LV dilation into the severe range (LV end-diastolic dimension > 65 mm) in three studies [14]. 

### 6.2. Surgical Management

**Figure 4:**
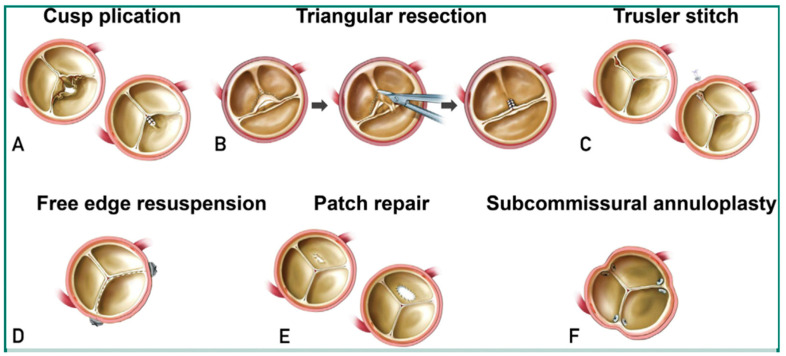
Aortic valve repair techniques. A prolapsed cusp is repaired with central plication (**A**), triangular resection (**B**), Trusler stitch (**C**), or free-edge resuspension (**D**). A perforated cusp is repaired with patch closure using pericardium (**E**). Annular dilation of aortic valve causing central regurgitation is repaired with plication stitches placed in the aortic wall at each commissure (**F**). (Reproduced with permission from Yang LT, Michelena HI, Maleszewski JJ, et al. Contemporary Etiologies, Mechanisms, and Surgical Approaches in Pure Native Aortic Regurgitation. *Mayo Clin. Proc.* [11].

Historically the definitive therapy has been surgery with aortic valve replacement or repair. Preoperative TEE with morphologic and functional classification of leaflet motion (Class I-III) with surgical anatomic inspection has been shown to be a predictor of success of “valve-conserving” surgical procedures [13]. The goal of surgical repair is to restore adequate leaflet coaptation surface and described repair techniques include cusp plication, triangular resection, Trusler stitch, free edge resuspension, patch repair, and subcommissural annuloplasty (Figure 4) [11]. Surgical replacement is principally performed with bioprosthetic and mechanical valves. Use of pulmonary autografts is considered in young patients with anatomy that is suboptimal for repair. Root repair techniques include STJ remodeling, root remodeling, and valve implantation within a vascular graft [57]. Patients with an aneurysmal aortic root or ascending aorta in combination with aortic regurgitation undergo the Bentall or modified Bentall procedure with a composite graft. 

Intervening on valvular heart disease before symptom development may prevent irreversible LV remodeling and improve clinical outcomes [43,58]. Asymptomatic AR is associated with increased mortality and morbidity [59] and current guidelines give a Class I level of recommendation for intervention in asymptomatic patients if the LV is markedly dilated (LV end-systolic [ES] dimension >50 mm or >25 mm/m^2^). However, growing evidence shows that poor outcomes are associated with a smaller LVES (and not end-diastolic) dimension of >20 mm/m^2^ [60,61,62]. Lower-indexed LVES dimensions could reduce the overall survival penalty seen for women related to late referral for intervention [63] particularly since women appear to exhibit a blunted LV dilatation response to increasing severity of AR [64]. 

## 7. Transcatheter Aortic Valve Replacement in Aortic Regurgitation

Historically there has been undertreatment with surgery of patients with significant AR. The Euro Heart Survey on Valvular Heart Disease showed a precipitous drop in the percentage of patients treated with a reduced LVEF and significant AR (2.7% of patients with LVEF <30%, 21.8% of patients with LVEF 30–50%) [65]. By current guidelines, TAVR can be considered only in selected patients at high or prohibitive surgical risk. If a patient is a surgical candidate with isolated severe AR, TAVR is a Class III recommendation [14]. Off-label TAVR with transcatheter heart valves designed for AS is frequently complicated by valve migration and significant paravalvular regurgitation due to the absence of valvular calcification needed for anchoring and aortic annular dilation [66]. To meet the clinical need, there are ongoing efforts to develop dedicated transcatheter heart valves that address the specific requirements for the treatment of pure AR. The JenaValve Trilogy Heart Valve System (JenaValve Technology, Inc., Irvine, CA, USA) is the first transfemoral device that has received CE mark approval for AR and AS treatment in Europe. Initial CE approval was based on the use of a transapical JenaValve device which showed a high procedural success rate of 89.6%, perioperative stroke rate of 3%, need for permanent pacemaker of 9.1%, and up to trace paravalvular leak in 86.4% of patients [67,68]. The JenaValve Trilogy design includes a self-expanding nitinol frame, porcine pericardial leaflets, and locators which attach to the native leaflets to aid with anchoring and sealing. The prosthesis clips onto the native leaflets which provides physiologic orientation, and anchoring is independent of native valve calcification. In the U.S., enrollment of 180 patients in the ALIGN-AR trial (NCT04415047) was completed in August 2022 using the TF JenaValve system and the one-year outcomes of this study are currently awaited. The J-Valve (Suzhou JieCheng Medical Technology Co., Ltd., Suzhou, China) is a self-expanding porcine tissue valve on a nitinol frame with three U-shaped anatomically oriented claspers developed to treat either AS or pure AR. The J-Valve was initially delivered transapically and the J-Valve TF Delivery System (JC Medical, Inc., Eatontown, NJ, USA) was developed to allow for transfemoral deployment. Two-year outcomes of the transapical Transcatheter Aortic Valve Implantation with J-Valve [57,69] showed a high success rate, and a high rate of improvement in heart failure symptoms. In patients treated with pure AR, at two years all-cause mortality was 11.6% and not significantly different from patients treated for AS. Additional trials with use of the J-valve are underway (NCT03876964, NCT05580952). 

## 8. Future Directions

The AHA/ACC and the ESC/EACTS valvular guidelines use parameters for intervention (symptoms, LVEF, LV dimensions) which are supported by outcomes data [14,70,71]. For the foreseeable future TTE will likely remain the primary tool for following and determining the timing of intervention. Further work is needed to determine which additional tools may provide prognostic value and may be integrated into the decision-making process in the future. In a recent multicenter prospective study evaluating CMR quantitative thresholds and outcomes in asymptomatic patients with moderate or severe AR with preserved LVEF, Malahfij et al. found that indexed LVES volume and indexed LV end-diastolic volume outperformed indexed LV end-systolic diameter [72]. Advanced imaging techniques like strain and myocardial work indices may become more commonly used in clinical practice. In addition to standard stress testing, cardiopulmonary exercise testing and cardiac markers may help in the management of asymptomatic patients. 

## 9. Conclusions

As outlined, the diagnosis, grading of severity, and management of AR requires a multimodality and multispecialty approach. This may be best guided by a Multidisciplinary Heart Valve Team [14,71] and consideration should be given to referral to a Primary (Level II) Valve Center or Comprehensive (Level 1) Valve Center [14]. A full armamentarium including an array of surgical and transcatheter treatment options will be needed to address the increasing clinical needs of an aging global population with AR. The future is bright in this respect with active research in novel transcatheter options to treat patients with an elevated surgical risk.

## Figures and Tables

**Table 1:** Primary etiologies of aortic regurgitation: Primary causes of aortic regurgitation (AR) can be categorized as degenerative, inflammatory, infectious, due to trauma, tissue disruption, iatrogenic, or congenital. Functionally there can be leaflet prolapse, restriction of leaflet motion, leaflet retraction, fenestration, or perforation (after Zoghbi WA, Adams D, Bonow RO, et al. Recommendations for Noninvasive Evaluation of Native Valvular Regurgitation: A Report from the American Society of Echocardiography Developed in Collaboration with the Society for Cardiovascular Magnetic Resonance. *J Am Soc Echocardiogr.* [10]).

Mechanism	Etiologies
Leaflet Abnormalities-Congenital	Bicuspid, unicuspid, or quadricuspid aortic valve Ventricular septal defect
- Acquired	Senile calcification Infective endocarditis Rheumatic disease Radiation-induced valvulopathy Toxin-induced valvulopathy; anorectic drugs, 5-hydroxtryptamine (carcinoid)
Aortic root abnormalities-Congenital/Genetic	Annuloaortic ectasia Connective tissue disease: Loeys–Deitz, Ehlers–Danlos, Marfan syndrome, osteogenesis imperfecta
- Acquired	Idiopathic aortic root dilation Systemic hypertension Autoimmune disease: systemic lupus erythematosus, ankylosing spondylitis, Reiter’s syndrome Aortitis: syphilitic, Tayayasu’s arteritis Aortic dissection Trauma

**Table 2:** **Grading Severity of Aortic Regurgitation:** Quantitative, semi-quantitative and qualitative techniques should be integrated when grading the severity (after Zoghbi WA, Adams D, Bonow RO, et al. Recommendations for Noninvasive Evaluation of Native Valvular Regurgitation: A Report from the American Society of Echocardiography Developed in Collaboration with the Society for Cardiovascular Magnetic Resonance. *J. Am. Soc. Echocardiogr.* [10]).

AR SEVERITY CLASSES	Mild Grade (1 or 1+)	Mild-to-Moderate (Grade 2 or 2+)	Moderate-to-Severe (Grade 3 or 3+)	Severe (Grade 4 or 4+)
QUALITATIVE PARAMETERS				
Aortic valve morphology	Normal/Abnormal	Normal/Abnormal	Abnormal/Prolapse/Moderate coaptation defect	Abnormal/Flail/Large coaptation defect
Color flow AR jet width ^$^	Small in central jets	Intermediate	Intermediate	Large in central jet, variable in eccentric jets
Color flow proximal convergence	None or very small	Dense	Intermediate	Large
CW signal of AR jet	Incomplete/Faint	Intermediate	Dense	Dense
Diastolic flow reversal in descending aorta ^#^	Brief, early diastolic reversal	Intermediate	Holodiastolic flow reversal (end-diastolic velocity 10 to <20 cm/s)	Holodiastolic flow reversal (end-diastolic velocity ≥ 20 cm/s)
Diastolic flow reversal in abdominal aorta ^#^	Absent	Absent	Present	Present
SEMI-QUANTITATIVE PARAMETERS				
VC width (mm)	< 3	3–6	3–6	>6
Jet width/LVOT diameter (%)	<25	25–45	46–64	≥65
Jet CSA/LVOT CSA (%)	<5	5–20	21–59	≥60
Pressure half-time (ms) ^#, £^	>500	Intermediate, 500 to 200	Intermediate, 500 to 200	<200
QUANTITATIVE PARAMETERS				
EROA (mm^2^) R Vol (ml) RF (%)	<10 <30 <30	10–19 30–44 30–39	20–29 45–59 40–49	≥30 ≥60 ≥50

AR: aortic regurgitation; CSA: cross-sectional area; CMR: Cardiac magnetic resonance; CW: continuous-wave; LA: left atrium; EROA: effective regurgitant orifice area; LV: left ventricle; RF: regurgitant fraction; R Vol: regurgitant volume; VC: vena contracta. ^$^ At a Nyquist limit of 50–60 cm/s. ^£^ Pressure half-time is shortened with increasing LV diastolic pressure, vasodilator therapy, and in patients with a dilated compliant aorta or lengthened in chronic AR. ^#^ These parameters are influenced by LV and aortic compliance. Hence, low transvalvular end-diastolic aorta to LV pressure gradient due to concomitant moderate/severe LV diastolic dysfunction may lead to false positive results. The high dependency of aortic flow reversal on aortic compliance considerably limits the utility of this parameter in the elderly population. These parameters are also influenced by chronotropy. Unless for other reasons, the LV size is usually normal in patients with mild AR. In acute severe AR, the LV size is often normal. Accepted cut-off values for non-significant LV enlargement: LV end-diastolic diameter < 56 mm, LV end-diastolic volume < 82 mL/m^2^, LV end-systolic diameter < 40 mm, LV end-systolic volume < 30 mL/m^2^.

## Supplementary Materials

The following supporting information can be downloaded at: https://drive.google.com/file/d/1fPm1uBO0LTRbhBWk9r12yJQHTboQj33N/view?usp=drive_web (accessed on 20 August 2023), Video S1: Transesophageal echocardiogram short axis view with biplane imaging through the left coronary cusp which is flail (A). Long axis imaging of the aortic valve with color showing eccentric, anteriorly directed regurgitation (B); Video S2: Transesophageal echocardiogram short axis view of a bicuspid aortic valve (Sievers type 0) 2D imaging (A) and with color (B), and biplane imaging with color showing eccentric aortic regurgitation (C); Video S3: Transesophageal echocardiogram short axis view of a quadricuspid valve without (A) and with color (B); Video S4: Transesophageal echocardiogram long axis view of tricuspid aortic valve with aortic dissection (Stanford Type A) (A). Loss of aortic root integrity with leaflet prolapse with aortic regurgitation seen on biplane imaging with color (B); Video S5: Transesophageal echocardiogram biplane imaging of an aortic valve with rheumatic involvement (leaflet doming, commissural fusion and nodular thickening) (A). Short axis aortic valve imaging with biplane showing moderate eccentric regurgitation; Video S6: Transesophageal echocardiogram imaging of an aortic valve with tubular dilation of the ascending aorta and effacement of the sinotubular junction. Biplane imaging of the aortic valve (A) and with color (B). 3D imaging showing a central coaptation gap (C).

## Abbreviations

AR	Aortic regurgitation
AS	Aortic stenosis
CMR	Cardiac MRI
CT	Computed tomography
ES	End-systolic
LV	Left ventricle/ventricular
LVEDP	Left ventricular end-diastolic pressure
MVAD	Mixed aortic valve disease
SAVR	Surgical aortic valve replacement
STJ	Sinotubular junction
TAVR	Transcatheter aortic valve replacement
TTE	Transthoracic echocardiogram
TEE	Transesophageal echocardiogram
